# Mitochondrial proteome of mouse oocytes and cisplatin-induced shifts in protein profile

**DOI:** 10.1038/s41401-021-00687-4

**Published:** 2021-05-20

**Authors:** Na Zhang, An-di Sun, Si-man Sun, Rui Yang, Yan-yan Shi, Qi-long Wang, Xin-yu Li, Ji-hong Ma, Wei Yue, Bing-teng Xie, Jie Qiao, Mo Li

**Affiliations:** 1 Center for Reproductive Medicine, Department of Obstetrics and Gynecology, Peking University Third Hospital, Beijing, 100191 China; 2 National Clinical Research Center for Obstetrics and Gynecology (Peking University Third Hospital), Beijing, 100191 China; 3 Key Laboratory of Assisted Reproduction (Peking University), Ministry of Education, Beijing, 100191 China; 4 Beijing Key Laboratory of Reproductive Endocrinology and Assisted Reproductive Technology, Beijing, 100191 China; 5 Research Center of Clinical Epidemiology, Peking University Third Hospital, Beijing, 100191 China

**Keywords:** mitochondrial proteome, APEX2, proximity labeling, mouse oocyte, cisplatin

## Abstract

Mitochondria are essential organelles that provide energy for mammalian cells and participate in multiple functions, such as signal transduction, cellular differentiation, and regulation of apoptosis. Compared with the mitochondria in somatic cells, oocyte mitochondria have an additional level of importance since they are required for germ cell maturation, dysfunction in which can lead to severe inherited disorders. Thus, a systematic proteomic profile of oocyte mitochondria is urgently needed to support the basic and clinical research, but the acquisition of such a profile has been hindered by the rarity of oocyte samples and technical challenges associated with capturing mitochondrial proteins from live oocytes. Here, in this work, using proximity labeling proteomics, we established a mitochondria-specific ascorbate peroxidase (APEX2) reaction in live GV-stage mouse oocytes and identified a total of 158 proteins in oocyte mitochondria. This proteome includes intrinsic mitochondrial structural and functional components involved in processes associated with “cellular respiration”, “ATP metabolism”, “mitochondrial transport”, etc. In addition, mitochondrial proteome capture after oocyte exposure to the antitumor chemotherapeutic cisplatin revealed differential changes in the abundance of several oocyte-specific mitochondrial proteins. Our study provides the first description of a mammalian oocyte mitochondrial proteome of which we are aware, and further illustrates the dynamic shifts in protein abundance associated with chemotherapeutic agents.

## Introduction

The decline in oocyte quality leads to fertilization failure and significantly poor reproductive outcomes [1]. Increasing evidence has shown that quality oocytes and successful fertilization require both cytoplasmic and nuclear maturation [2]. Genomic integrity, regular epigenetic modifications, and meiotic competence are representative features of nuclear maturation [3–5], while cytoplasmic maturation involves the storage of maternal factors and reorganization of diverse organelles such as the mitochondria, endoplasmic reticulum, Golgi apparatus, and cortical granules [6–8]. Among these organelles, mitochondria play essential roles in oocyte maturation. Because of the huge volume, female germ cells contain a larger number of mitochondria than in somatic cells. Moreover, mitochondria produce energy for the whole oocyte by oxidative phosphorylation, and they move to areas of high energy consumption during oocyte maturation [9]. As an essential uniparental-inherited organelle, oocyte mitochondria contribute all mitochondrial components for fertilized oocytes and early embryos. In contrast, the mitochondrial proteins in sperm are subject to ubiquitination upon entry into the oocyte, leading to their subsequent proteasomal degradation and autophagy [10, 11]. Oocyte mitochondria are therefore required to support oocyte maturation and early embryonic development. In addition, numerous heritable mitochondrial diseases originate from either mutations in mtDNA genes or dysfunction of oocyte mitochondrial proteins [12–14]. However, due to the rarity of oocyte samples and the technical limitations involved, systematic protein profiling has not yet been conducted for oocyte mitochondria, which would be highly informative for basic understanding oocyte mitochondrial function, especially for live oocytes.

As an emerging protein labeling technology, engineered ascorbate peroxidase (APEX2)-mediated reaction can achieve biotinylation of the surrounding proteins (i.e., Tyr, Trp, His, and Cys biotinylation) within 1 min. Besides, its small labeling radius and amenability to tyramide signal amplification make microscale reactions possible [15, 16]. Recent studies show the advantages of APEX labeling in subcellular regions in proliferating cell lines [17, 18]. However, the application of APEX in non-proliferating cells, especially in germ cells, is absent. Due to the long period of arrest at the germinal vesicle (GV) stage before meiotic resumption, the quality of GV-stage oocytes is closely associated with subsequent oocyte maturation and successful fertilization. Thus, establishing a comprehensive assessment system, such as a system based on the mitochondrial quality of GV oocytes, would be valuable. Besides, with the increase of tumor incidence in women, chemotherapeutic treatments give rise to substantial side effects on female germ cells [19, 20]. However, to what extent and what kinds of cellular components are impaired by chemotherapeutic treatments are far from clear.

In the current study, our objective was to decipher the proteome of mitochondria in live GV oocytes of mice and explore alterations in the mitochondrial proteome after chemotherapeutic treatment with cisplatin. Utilizing engineered ascorbate peroxidase (APEX2) technology [16, 17], we established a mitochondria-APEX2 (Mito-APEX2) reaction to selectively biotinylate mitochondrial proteins in living oocytes with high spatial specificity. As a result, we identified a total of 158 proteins in oocyte mitochondria, which include intrinsic components of mitochondrial structure and are mainly involved in “cellular respiration”, “ATP metabolic”, and “mitochondrial transport” processes, among others. Following low dose treatment of cisplatin, we found that oocyte mitochondrial function was impaired, with increased ROS levels and lower mitochondrial membrane potential. In addition, the abundance of mitochondrial proteins associated with several biological processes (i.e., oxidation reduction process) were altered. Thus, this work provides a mitochondrial proteomics resource for expanded research in mouse oocyte models and provides candidate proteins to use for assessing clinical oocyte quality during chemotherapeutic treatments.

## Materials and methods

### Oocyte collection and culture

This study was reviewed and approved by the Ethics Committee of Peking University Third Hospital (No. LA2018256), and all mice were kept and treated following the policies promulgated by the Ethics Committee on the use of animals in research. Female ICR mice at 6–8 weeks of age were sacrificed by cervical dislocation 44–46 h after intraperitoneal injection of 10 IU pregnant mare serum gonadotropin (PMSG, NSH, Ningbo, China). Immature oocytes displaying clear germinal vesicles (GVs) were collected and cultured in M2 medium (Sigma-Aldrich, MO, USA) supplemented with 2.5 μM milrinone (Sigma-Aldrich) to maintain the prophase of meiosis I arrest. For cisplatin treatment, immature oocytes were cultured in M16 medium (containing 2.5 μM milrinone) with low (1 μM) or high (20 μM) dosages of cisplatin (Sigma-Aldrich) for 4 h, then oocytes were washed and cultured in fresh M16 (Sigma-Aldrich) medium until they reached the MII stage. The matured oocytes were fertilized by intracytoplasmic sperm injection and cultured in KSOM (Sigma-Aldrich) at 37 °C in a 5% CO_2_ atmosphere.

### Plasmid construct and cRNA preparation

To guide engineered ascorbate peroxidase (APEX2) to the oocyte mitochondrial matrix, the APEX2 sequence was fused to the coding sequence (CDS) of *Txn2* and subcloned into pcDNA3 with an enhanced green fluorescent protein (EGFP) tag to construct THIOM-APEX2-EGFP plasmid. The coding regions of mouse *Hsp90aa1, Eef2, Rack1 *and *Txn2* were amplified through PCR and then subcloned into pcDNA3-EGFP plasmids. For fusion protein expression, *Hsp90aa1* and actin were cloned into pGEX-4T-1. For cRNA preparation, plasmids were linearized with a restriction enzyme targeting a site downstream of the EGFP tag. A T7 or SP6 mMESSAGE mMACHINE Kit (Thermo Fisher Scientific, MA, USA) was used to synthesize cRNAs in vitro with the linearized plasmids as templates. Then, the cRNAs were added to poly(A) tails by a poly(A) tailing kit (Thermo Fisher Scientific) and purified. Finally, poly(A)-tailed RNAs were eluted with nuclease-free water and stored in aliquots at −80 °C.

### Microinjection of cRNAs

To perform microinjection, cRNA solution was loaded into glass micropipettes at the concentration of at least 1 mg/mL. Then, cRNAs were injected into GV oocyte cytoplasm using a FemtoJet Microinjector (Eppendorf, Hamburg, Germany) and micromanipulators (Narishige, Tokyo, Japan) on the stage of an inverted microscope. The volume of cRNA injected into each oocyte was 7–10 pL. Manipulation was accomplished in drops of M2 medium, after which oocytes were maintained in M16 medium with 2.5 μM milrinone for 3 h to allow translation of protein. The oocytes were then washed and cultured in fresh M16 medium drops.

### NIH3T3 cells culture and plasmid transfection

NIH3T3 cells were cultured in Dulbecco’s Modified Eagle’s Medium (DMEM, Gibco, Carlsbad, USA) containing 10% fetal bovine serum (FBS, Biological Industries, Cromwell, USA) and 1% penicillin-streptomycin. Cells were cultured in a humidified atmosphere of 5% CO_2_ and 95% air at 37 °C. NIH3T3 cells were seeded in glass-bottom cell culture dishes. JetPRIME Transfection Reagent (Polyplus-transfection S.A., Strasbourg, France) was used to transfect plasmids (*Hsp90aa1-, Eef2-, Rack1-* and *Tfam*-pcDNA3-EGFP) into cells, respectively. According to the manufacturer’s instructions, 2 µg DNA was diluted into 200 µL jetPRIME® buffer and mixed by vortexing, then 4 µL jetPRIME® was added, vortexed, and incubated for 10 min at room temperature to make transfection mix. When the cells reached ~70% confluence, transfection reagent mix (100 µL/well) was evenly added to the cell medium and replaced by fresh medium 4 h after transfection. Expression of the transfected plasmid of cells was confirmed by GFP 24 h later.

### Immunofluorescence and confocal microscopy

For mitochondria staining, living oocytes or cells were stained with 200 nM MitoTracker 633 dye (Thermo Fisher Scientific) in an atmosphere of 5% CO_2_ at 37 °C for 30 min. After washed with fresh medium, oocytes were fixed in 4% paraformaldehyde in PBS (pH 7.4) for 30 min and permeabilized in 0.5% Triton X-100 for another 30 min at room temperature. Then, oocytes were blocked with 1% bovine serum albumin-supplemented PBS for 60 min. For Alexa Fluor® 555 streptavidin (Thermo Fisher Scientific) staining, oocytes were incubated with Alexa Fluor® 555 streptavidin (1:100) for 2 h at room temperature. DNA was stained with Hoechst 33342 (10 μg/mL) for 30 min. Finally, oocytes were washed three times followed by mounting on glass slides, and observed using a confocal laser scanning microscope at ×63/1.40 (Carl Zeiss, Jean, Germany).

### Determination of ROS levels

Oxygen Species Assay Kit (Beyotime, Shanghai, China) and MitoSOX Red (Thermo Fisher Scientific) were used to assess ROS levels in oocytes. Dichlorofluorescein (DCFH) is an oxidation-sensitive fluorescent probe. Oocytes were treated with or without cisplatin (1 or 20 μM) for 4 h and then incubated in M2 medium with 10 μM DCFH diacetate (DCFHDA) for 30 min at 37 °C. All three groups of oocytes were washed separately three times in fresh medium and images were captured on a scanning confocal microscope. For MitoSOX staining, cisplatin treated and non-treated oocytes were incubated in M2 medium containing 5 μM MitoSOX Red for 10 min at 37 °C in the dark. After washing three times in fresh medium under low light, oocytes were imaged under a Carl Zeiss 710 confocal microscope. In each experiment, fluorescence signals were acquired by confocal microscopy with the same scanning settings. ImageJ software (NIH Image, Bethesda, MD) was used to quantify fluorescence intensity as previously reported [21]. Briefly, fluorescence channels of the confocal images were separated and converted to 8-bit images, and then a threshold was set for each channel of fluorescence. The mean fluorescence intensity of thresholded fluorescence images of different groups was calculated using the measurement function in ImageJ software.

### Western blot

Oocytes were lysed in RIPA buffer containing 1× protease inhibitor. Protein samples were boiled and separated by SDS-PAGE and then electrically transferred onto PVDF membrane. After transfer, the membranes were blocked in 1× TBST containing 5% skimmed milk or 3% BSA for 2 h at room temperature, followed by incubation with indicated primary antibodies overnight at 4 °C. The following antibodies were used: anti-actin antibody (Thermo Fisher Scientific), anti-EF2 antibody (Cell Signaling Technology, MA, USA), anti-HS90A antibody (Abcam, MA, USA), anti-RACK1 antibody (Abcam) and anti-COX IV antibody (Cell Signaling Technology). After washing in 1× TBST for three times, the membranes were incubated with 1:1000 dilution of HRP-conjugated secondary antibody for 1 h. For biotin-labeled proteins, streptavidin-HRP conjugate (Thermo Fisher Scientific) was used to incubating membranes after blocking. Finally, protein bands were visualized by an enhanced chemiluminescence detection system.

### Microscale thermophoresis

Microscale thermophoresis (MST) was performed according to the previous work as described [22]. In brief, GST-HS90A, GST-actin and GST were expressed in *Escherichia coli* and purified under standard procedures. GST-HS90A, HSPE1 (Proteintech, Chicago, USA), GST-actin or GST were labeled with a RED-NHS protein labeling kit (NanoTemper, Munich, Germany). The protein was then incubated at a constant concentration (10–100 nM) with twofold serial dilutions of cisplatin in MST-optimized buffer (50 mM Tris-HCl, pH 7.4, 150 mM NaCl, 10 mM MgCl_2_, 0.05% Tween-20). Equal volumes of binding reactions were mixed by pipetting and incubated for 15 min at room temperature. Mixtures were enclosed in standard-treated or premium-coated glass capillaries and loaded into the instrument (Monolith NT.115, NanoTemper, Germany). Measurement protocol times were as follows: fluorescence before 5 s, MST on 30 s, fluorescence after 5 s, and delay 25 s. For all the measurements, 200-1000 counts were obtained for the fluorescence intensity. The measurement was performed at 20% and 40% MST power. *F*_norm_ = *F*_1_/*F*_0_ (*F*_norm_: normalized fluorescence; *F*_1_: fluorescence after thermodiffusion; *F*_0_: initial fluorescence or fluorescence after *T*-jump). *K*_*d*_ values were determined with the NanoTemper analysis tool.

### Separation of cytosolic and mitochondrial fractions

Cytosolic and mitochondrial fractions were separated using mitochondria isolation kit (Thermo Fisher Scientific) according to the manufacturer’s instructions. Briefly, oocytes were treated with 0.5% pronase (Sigma-Aldrich) to remove the zona pellucida. NIH3T3 cells (1 × 10^7^) or oocytes (2000) were collected and lysed in mitochondria isolation reagent A on ice. Then mitochondria isolation reagent C was added in the lysate, and it was mixed and centrifuged for 10 min at 700 × *g*. Next, the supernatant was transferred into a new tube and centrifuged at 12,000 × *g* for 15 min at 4 °C. The supernatant (cytosol fraction) was transferred to another tube and the pellet containing the isolated mitochondria was lysed in RIPA buffer, thus cytosol and mitochondrial fractions were separated.

### APEX2-mediated biotinylation

Oocytes expressing THIOM-APEX2-EGFP (reaction group) or THIOM-EGFP (control group) were collected and incubated in medium drops with 500 μM biotin-phenol for 30 min. The reaction was initiated when the oocytes were transferred to media drops containing 1 mM H_2_O_2_ using a mouth pipette. One minute later, the reaction was quenched by quickly transferring the oocytes to a “quencher solution” (10 mM sodium ascorbate, 10 mM sodium azide, and 5 mM Trolox in DPBS) and washed for three times. For cisplatin treated group, oocytes expressing THIOM-APEX2-EGFP were incubated in media with 1 μM cisplatin for 4 h followed by Mito-APEX2 reaction. The reacted oocytes were subsequently collected for mass spectrometry or Western blot.

### Enrichment of biotinylated proteins

The reacted oocytes were lysed in RIPA buffer containing 1× protease inhibitor cocktail. For streptavidin enrichment and elution of biotinylated proteins, streptavidin-coated magnetic beads (Thermo Fisher Scientific) were firstly washed twice with RIPA lysis buffer. Each Sample (in 0.2% SDS RIPA buffer) was incubated in 500 μL RIPA buffer added with 10 μL streptavidin-coated magnetic beads slurry, rotating at room temperature for 2 h to bind biotinylated proteins. And then the flowthrough after enrichment was removed, and the beads were subsequently washed with 1 mL RIPA lysis buffer for two times, followed by once washing with 1 mL 1 M KCl, once washing with 1 mL 0.1 M Na_2_CO_3_, once washing with 1 mL 2 M urea in 10 mM Tris-HCl (pH 8.0), and twice washing with 1 mL RIPA lysis buffer. Finally, biotinylated proteins were eluted by boiling the beads in 50 μL protein loading buffer supplemented with 20 mM DTT and 2 mM biotin.

### Sample preparation

Proteins sample from oocytes (1500 oocytes/sample) for mass spectrometry were prepared according to previous studies [17]. Biotinylated proteins eluted from streptavidin beads were run on SDS-PAGE. Following Coomassie Brilliant Blue staining and destaining, the lane of each sample on gels was divided into several target strips and digested with trypsin respectively. Then the strips were mixed for mass spectrometry.

### LC-MS/MS analysis

Peptide mixtures were analyzed on an Orbitrap Fusion Lumos (Thermo Fisher Scientific) mass spectrometer interfaced with an Easy-nLC 1200 nanoflow liquid chromatography system (Thermo Fisher Scientific) with a Nono Spray Ionization (NSI) in positive ion polarity. Samples were dissolved with 15 μL of mobile phase A (0.1% formic acid in water), pickup 5 μL to loop ring with auto-sampler, loaded to a homemade trap column (2 cm × 100 μm) packed with C18 reverse-phase resin (particle size, 3 μm; pore size, 120 Å; Dr.Maisch, Germany) at a maximum pressure of 280 bar with 12 μL of solvent A, then separated on a 150 μm × 15 cm silica microcolumn (homemade, particle size, 1.9 μm; pore size, 120 Å; Dr.Maisch, Germany) with a gradient of 11–100% mobile phase B (80% acetonitrile and 0.1% formic acid) at a flow rate of 600 nL/min for 30 min. The gradient elution conditions were: 11% to 41% mobile phase B for 25 min; 41 to 100% for 1 min; 100% for 4 min. A nono spray ionization source was used to ionize the eluted peptides and then they were analyzed on an Orbitrap Fusion Lumos mass spectrometer.

The MS analysis was performed in a data-dependent manner (DDA) with full scans (m/z 350–1550) acquired using an Orbitrap mass analyzer at a mass resolution of 120,000, and the automatic gain control (AGC Targets) was set to 5e5 with a maximum ion injection times of 50 ms. The most intense ions selected under top-speed mode were isolated in Quadrupole with a 1.6 m/z window and fragmented by higher-energy collisional dissociation (HCD) with a normalized collision energy of 32%, then detected in the Orbitrap at a mass resolution of 15,000, the automatic gain control (AGC Targets) for MS/MS was set to 3e4, maximum ion injection times were dynamic. The dynamic exclusion time was set as the 30 s, and isotope exclusion was enabled.

### Protein identification and quantification

Raw files were processed by Proteome Discoverer (Thermo Fisher Scientific, version 2.2), and searched against the mouse NCBI RefSeq protein database (2017-11-01) using the SEQUEST HT search engine with a percolator. The search parameters used were as follows: the mass tolerance of the precursor ions was set to 20 ppm, the tolerance of the productions was set to 0.05 Da. Up to two missed cleavages were allowed for trypsin digestion. Carbamidomethylation of cysteine, Oxidation of methionine, and acetylation of N-terminal protein were set as variable modifications. Searches used a reversed sequence decoy strategy to control peptide false discovery and identifications were validated by Percolator software, false discovery rate (FDR) was set to 0.01 for proteins, peptide, and peptide-spectrum matches (PSMs). The sum of all peptides were used for protein quantifications. Two replicates of reaction (oocytes expressing THIOM-APEX2-EGFP), cisplatin treated (oocytes expressing THIOM-APEX2-EGFP and treated with cisplatin) and control (oocytes expressing THIOM-EGFP) experiments were conducted. We only kept protein abundance values are positive in two replicates. Proteins with their average quantitative abundance values in reaction groups twofolds higher than those in control groups were identified as oocyte mitochondrial proteins. Proteins with their quantitative abundance values in reaction groups twofolds higher or lower than those in cisplatin-treated groups in two replicates were screened out as differential proteins.

### Bioinformatics analysis and data availability

Heatmaps of the oocyte mitochondrial protein expression profiles and chord plot were generated using R packages. For the objective imputation of missing values, the deterministic minimum imputation (MinDet) method was adopted. Gene Ontology (GO) analysis of oocyte mitochondrial proteins was conducted using R-cluster Profiler. The representative GO terms were shown and *P* values were adjusted. Gene set enrichment analysis (GSEA, https://www.gsea-msigdb.org/gsea/index.jsp) was managed to identify concordant differences between cisplatin-treated or non-treated oocyte mitochondrial proteome, the core enriched proteins were shown by leading edge analysis [23]. The mass spectrometry proteomics data have been deposited to the ProteomeXchange Consortium (http://proteomecentral.proteomexchange.org) via the iProX partner repository [24] with the identification No. PXD024777 (for ProteomeXchange) and IPX0002895000 (for iProX).

### Statistical analysis

Means and standard deviations were plotted, and Student’s *t*-test was used to compare the data. The statistical differences were considered significant when the *P* < 0.05 (*). All experiments were performed in triplicates unless indicated otherwise.

## Results

### Targeting active APEX2 to the mitochondrial matrix of living oocytes

**Fig. 1:**
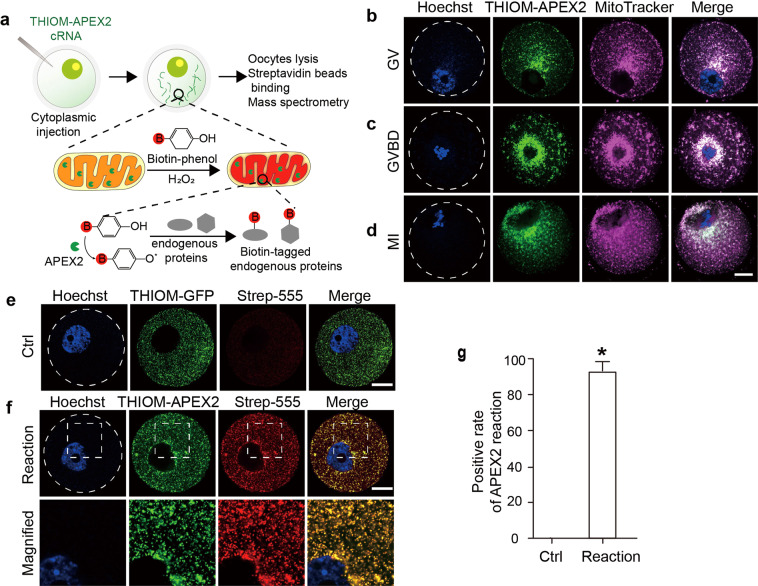
APEX2 targeting to oocyte mitochondria. **a** Flow diagram of APEX2 reaction in mouse GV oocytes. The linearized cRNA of THIOM-APEX2-EGFP was microinjected into oocytes at the GV stage arrested by milrinone. When the APEX2 fusion protein was expressed, the oocytes were incubated with biotin-phenol (BP) for 30 min, and the reaction was triggered by H_2_O_2_ for 1 min. After reaction quenching, the oocytes were immediately lysed for subsequent experiments. **b**–**d** Subcellular localization of THIOM-APEX2-EGFP during oocyte meiosis. After intracytoplasmic microinjection of linearized THIOM-APEX2-EGFP cRNA, the fluorescence signals of THIOM-APEX2-EGFP (green), MitoTracker (magenta), and DNA (blue) were detected. Oocytes at GV, GVBD, and MI stages are shown. **e**, **f** Confocal fluorescence images show the localization pattern of THIOM-APEX2-EGFP and the reaction labeled by streptavidin. Oocytes expressing THIOM-EGFP were used as a negative control. Magnified images show details of streptavidin-555 overlaid with THIOM-APEX2-EGFP. Dashed outline indicates the plasma membrane border of the oocyte. **g** Streptavidin positive rates in control and reaction groups. At least 100 oocytes from three independent experiments were counted for each group. The data represent the mean ± SD. *P < 0.05. Scale bar, 20 μm.

In this study, we began by targeting APEX2 to mitochondria using a bait protein to establish a specific reaction within the oocyte mitochondria. Based on previous reports, we selected 10 candidate bait proteins that are both highly expressed in mouse oocytes (RPKM > 50) and specifically localized in the mitochondria [25, 26] (Fig. S1). Among them, thioredoxin (THIOM, coded by *Txn2*) was well-characterized to be localized in the mitochondrial matrix and had the smallest molecular weight. We generated cRNA fusion constructs of APEX2-EGFP with THIOM and transformed them into oocytes via intracytoplasmic microinjection for expression and subsequent biotin-phenol labeling (Fig. 1a).

Imaging by confocal microscopy showed that THIOM-APEX2-EGFP was precisely localized in mitochondria, as confirmed by MitoTracker staining. THIOM-APEX2-EGFP was retained exclusively within the mitochondria during the whole oocyte maturation process, including GV, germinal vesicle breakdown (GVBD), and meiotic stages (Fig. 1b–d). To examine whether THIOM-APEX2 could be employed for mitochondrial proteomic labeling, we triggered the APEX2 reaction and detected the reaction rate in GV oocytes. Fluorescently labeled streptavidin was used to assess the localization of biotinylated proteins. The imaging results showed that biotinylated proteins overlapped tightly with THIOM-APEX2 within mitochondria in THIOM-APEX2 reacted oocytes, while no biotinylated proteins were detectable in THIOM-EGFP control oocytes (Fig. 1e, f). The positive reaction rate in the reacted group was ~92% (Fig. 1g). These results suggest that THIOM-APEX2 is catalytically active in oocytes and thus can potentially be used for the Mito-APEX2 reaction during oocyte maturation.

### Proteome profiling of oocyte mitochondria

**Fig. 2:**
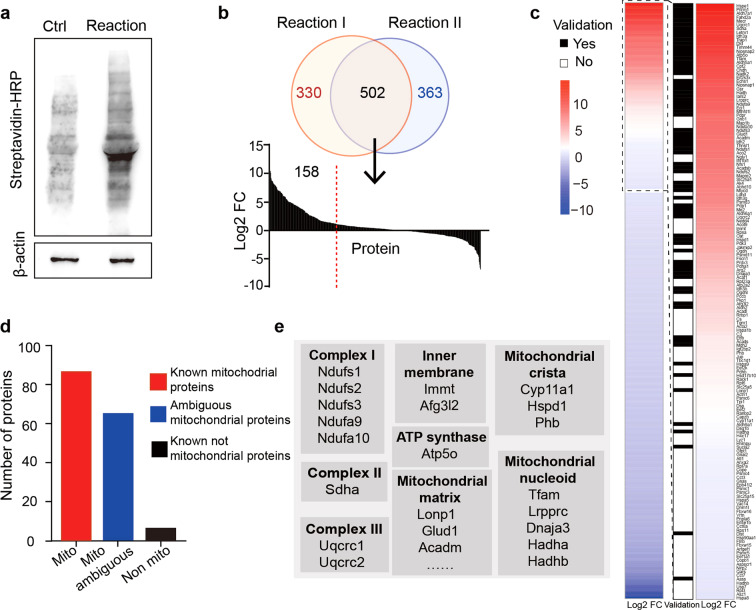
Mito-APEX2 reaction and proteomics analysis. **a** SDS-PAGE of cell lysates from Mito-APEX2 reaction detected by Western blot against biotin. Oocytes expressing THIOM-EGFP were used as negative controls. β-actin was used as a loading control. **b** Screening of APEX2 biotinylated proteins by liquid chromatography-tandem mass spectrometry (LC-MS/MS). Reaction I and Reaction II included proteins identified by LC-MS/MS in the reaction group of two independent experiments. Upper: Venn diagram showing the total number and overlap of proteins from Reaction I and II. Lower: Bar graph of log2 fold change (FC) of average quantitative values for proteins identified in both Reaction I and II. **c** Proteins identified in streptavidin affinity purification from the APEX2 oocytes were ranked in descending order by ratio of average quantitative values between the reaction and control groups. The intensity of the blue-red color represented the log2 FC between the reaction and control group. Black boxes indicate whether a protein was previously validated as a mitochondrial protein. The boxed inset shows the identified mitochondrial proteins (log2 FC >1). **d** Mitochondrial proteins subdivided by mitochondrial annotation using MitoMiner 4.0. Known mitochondrial proteins are plotted in red, known non-mitochondrial proteins are plotted in black, and ambiguous mitochondrial proteins are plotted in blue. **e** Distribution and roles of the known mitochondrial proteins in submitochondrial localization. Proteins located in mitochondrial inner membrane, matrix, and complexes I-V are listed.

After the Mito-APEX2 reaction was performed in THIOM-APEX2-EGFP expressing oocytes, the samples were lysed, and the proteins were separated by gel electrophoresis. Streptavidin blots showed that THIOM-APEX2-EGFP biotinylated a mass of endogenous proteins across a range of molecular weights in reacted oocytes, while few biotinylated proteins were observed in control oocytes, in which cRNA in absence of APEX2 (THIOM-EGFP) was injected (Fig. 2a). These results indicated successful capture of the mitochondrial proteome from mouse oocytes. Next, we generated proteomic data by nano LC-MS/MS (liquid chromatography tandem mass spectrometry) from the reaction groups and control groups (used for nonspecific deduction from reaction groups). Due to the numerical limitation of germ cells, which is unlike those of proliferating somatic cells, we performed two rounds of repeats, and included 1500 oocytes in each control and reacted group, i.e., 1500 × 2 = 3000 for the control groups, and 1500 × 2 = 3000 for the reaction groups. More than 800 proteins were detected in each of the two independent reaction groups and 502 proteins were found in both groups (Fig. 2b, Table S1, S2). Among the 502 proteins, 158 proteins were found with average quantitative abundances specific to or at least 2-fold higher (log_2_ FC > 1) in the reacted group than in the control groups (Fig. 2b, c); these proteins were identified as oocyte mitochondrial proteins (Table S3).

To confirm the specificity of the protein list, we compared our data to MitoMiner 4.0, an updated database of mitochondrial localized proteins [27]. Among the 158 proteins, 87 were annotated as ‘known mitochondrial’, 63 were annotated as “ambiguous”, and 8 were annotated as ‘known not mitochondrial’ (Fig. 2d), indicating a relatively high positive rate of the list. Furthermore, we compared our oocyte mitochondrial proteome to a previously reported mitochondrial proteome from somatic cells [17] and found that dozens of proteins were not recorded in the somatic mitochondrial proteome (Fig. 2c, black lines indicated overlapping proteins mapping to that study [17]), implying unique characteristics of the oocyte mitochondrial proteome. Moreover, our proteome provided broad coverage of the proteins in distinct compartments including the mitochondrial matrix, mitochondrial cristae, and respiratory chain complexes from complex I to complex V (Fig. 2e). These results suggested that Mito-APEX2 labeling enabled the identification of a comprehensive oocyte mitochondria-specific proteome.

### Characterization of oocyte mitochondrial proteins

**Fig. 3:**
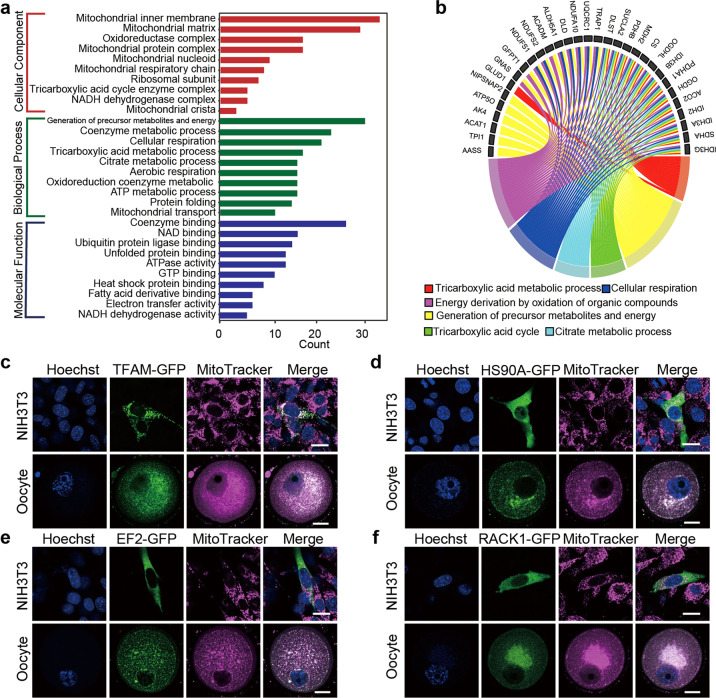
Characterization of oocyte mitochondrial proteome specificity. **a** Functional annotation and GO (gene ontology) enrichment analysis of identified mitochondrial proteins. Selected enriched GO-term categories for cellular components, biological process, and molecular function are shown based on significance value. The x-axis represents gene counts for each term. **b** Chord plot depicting the relationship between oocyte mitochondrial proteins and GO terms of biological process. **c**–**f** Validation of new oocyte mitochondrial proteins. Subcellular localization of three proteins (HS90A, EF2, and RACK1) in oocytes and NIH3T3 cells. Known mitochondrial protein TFAM was used as a positive control to confirm localization in mitochondria. Upper: NIH3T3 cells were transiently transfected with *Tfam-*, *Hsp90aa1-*, *Eef2-*, and *Rack1-*pcDNA3-EGFP plasmids, respectively. Fluorescence was detected at 24 h after transfection. Lower: cRNAs of *Tfam-*, *Hsp90aa1-*, *Eef2-*, and *Rack1-*EGFP were microinjected into oocyte cytoplasm and imaged using confocal microscopy. MitoTracker (magenta), DNA (blue). Scale bar, 20 μm.

To analyze the characteristics and functions of the oocyte mitochondrial proteome, all 158 proteins were subjected to Gene Ontology (GO) enrichment analyses. The GO terms enriched in cellular components covered nearly the whole spectrum of submitochondrial compartments, including ‘mitochondrial inner membrane’ (33 proteins), ‘mitochondrial matrix’ (29 proteins), ‘oxidoreductase complex’ (15 proteins), and ‘mitochondrial nucleoid’ (9 proteins) (Fig. 3a, Table S4). Since mitochondria serve as the source of cellular energy by generating adenosine triphosphate through oxidative phosphorylation, we hypothesized that biological processes involved in energy metabolism would be significantly enriched in our oocyte mitochondrial proteome. Indeed, GO enrichment analysis further showed that a series of energy metabolic processes were enriched among the proteins in our dataset, such as “generation of precursor metabolites and energy”, “coenzyme metabolic process”, “cellular respiration”, and “tricarboxylic acid metabolic process” (Fig. 3a, b). Molecular functional analysis of these proteins revealed enrichment for functions such as “NAD binding”, “unfolded protein binding”, and “ATPase activity” (Fig. 3a, Table S4). These analyses indicated that our mitochondrial proteome included a large range of proteins involved in multiple submitochondrial compartments and various biological processes clearly related to mitochondrial functions.

Mitochondria necessarily adjust their protein abundances depending on development-, tissue-, and even species-specific metabolic requirements [28]. To better understand these requirements as they pertain to oocytes, we focused on deeper exploration of the proteins that were enriched in several GO terms closely connected to mitochondrial function (Fig. 3a), but were not detected in the prior somatic mitochondrial proteome, thus indicating their status as newly found oocyte mitochondrial proteins. To verify the localization of these proteins in oocytes, we fused GFP to the C-terminus of three ambiguous proteins (HS90A, involved in ‘protein folding process’; EF2, involved in “GTP binding”; and RACK1, involved “ribosomal subunit”) and one known mitochondrial protein (TFAM). We microinjected cRNAs of these proteins with an EGFP tag into the oocyte. In parallel, we transfected these plasmids into the NIH3T3 mouse somatic cell line. TFAM, a well-known mitochondrial transcription factor, localized to mitochondria in both oocytes and NIH3T3 cells (Fig. 3c). Interestingly, HS90A was specifically expressed in oocyte mitochondria, as confirmed by MitoTracker staining, but was diffusely distributed in the NIH3T3 cell cytoplasm and did not exhibit mitochondrial localization (Fig. 3d). Similar results were observed in EF2 and RACK1 (Fig. 3e, f). Furthermore, the cytosolic and mitochondrial fractions of NIH3T3 cells and oocytes were separated and the expression of proteins was detected by Western blotting. Consistent with the imaging experiments, HS90A, EF2, and RACK1 mainly existed in mitochondria of oocytes, while meanly existed in cytosolic fraction of 3T3 cells (Fig. S2). These results demonstrated that certain proteins were specifically localized in mitochondria of oocytes, implying that we obtained an oocyte-specific mitochondrial proteome.

### Low dose of cisplatin impairs oocyte maturation and induces mitochondrial dysfunction

**Fig. 4:**
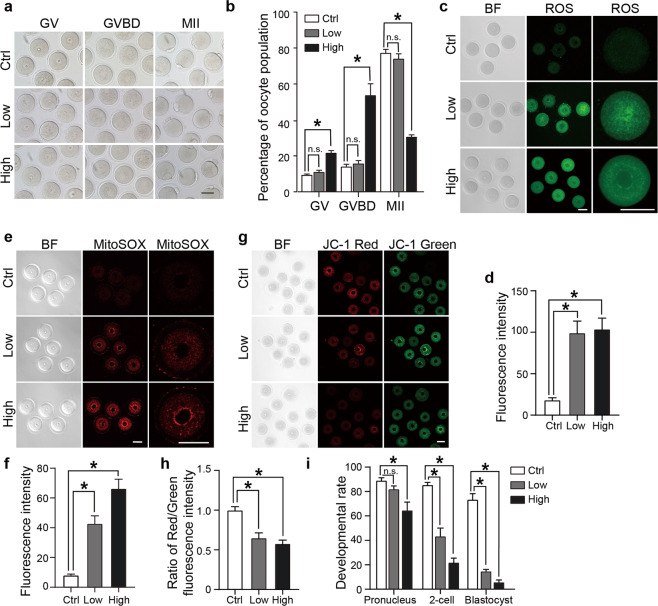
Effects of cisplatin on oocyte development and mitochondria. **a** Oocyte morphology during maturation with or without cisplatin treatment. Neither high-dose nor low-dose cisplatin changed oocyte morphology. **b** Rates of oocyte maturation with or without cisplatin treatment. High-dose cisplatin inhibited oocyte maturation. At least 100 oocytes from three independent experiments were counted for each group. **c**–**f** ROS assessment of oocytes with or without cisplatin exposure. **c** Representative images of cellular ROS levels detected by DCFH staining. **d** Fluorescence intensity was used to quantitatively measure cellular ROS levels. The histogram shows the fluorescence intensity of the DCFH signals. **e** Mitochondrial ROS levels were detected with MitoSOX in control and cisplatin-treated oocytes. **f** Fluorescence intensity of MitoSOX signals measured in control, low- and high-dose cisplatin-treated oocytes. **g**, **h** Mitochondrial membrane potential assessment of oocytes with or without cisplatin treatment. The oocytes were stained with the mitochondria-specific probe JC-1. Red and green fluorescence indicate J-aggregates and JC-1 monomers, respectively. The red: green fluorescence intensity ratio was used to indicate the mitochondrial membrane potential. **i** The effect of cisplatin treatment on early embryonic development. GV oocytes were treated with low and high dosages of cisplatin for 4 h and incubated with fresh culture medium for in vitro maturation. The mature oocytes were subjected to intracytoplasmic sperm injection followed by the assessment of early embryonic development at the pronucleus, 2-cell and blastocyst stages. The data represent the mean ± SD. *P < 0.05; ^ns^*P* > 0.05. Scale bar, 50 μm.

Anticancer therapy is often a cause of premature ovarian insufficiency and infertility since immature oocytes are extremely sensitive to the effects of chemotherapy. In particular, cisplatin is widely used for the treatment of several tumor types [19]. However, the extent and kinds of cellular components that are impaired by cisplatin treatment are far from clear. In addition, it is important to identify a safe dosage of cisplatin in terms of fertility preservation. The plasma cisplatin concentration of women under chemotherapy treatments ranges from 1 to 20 μM [29, 30]. Thus, we treated GV oocytes with high (20 μM) and low (1 μM) dosages of cisplatin, followed by detection of oocyte morphology, in vitro maturation and molecular phenotypes. Neither high-dose nor low-dose cisplatin-treated group caused morphological changes (Fig. 4a). The oocyte maturation rate in high-dose cisplatin treated group was significantly decreased (Fig. 4b), accompanied by an elevated level of ROS, which was assessed by MitoSOX and DCFH staining (Fig. 4c–f). In addition, the mitochondrial membrane potential (Δψm) was examined by JC-1 staining. JC-1 is a fluorescent ΔΨm reporter that forms J-aggregates and emits red fluorescence when mitochondrial membrane potential (ΔΨm) is high, whereas it remains a monomer and emits green fluorescence when ΔΨm is low. The decrease in the relative ratio of red to green fluorescence indicates a decreased ΔΨm [31]. Consistent with the ROS results, high-dose cisplatin treatment impaired mitochondrial membrane potential (Fig. 4g, h). Interestingly, although low-dose cisplatin treatment did not result in a change in the oocyte maturation rate, oocyte ROS levels were elevated (Fig. 4c–f), and the mitochondrial membrane potential was impaired (Fig. 4g, h). In addition, in the control group, the mitochondria were homogeneously distributed throughout the oocytes. However, upon treatments with low- or high-dose cisplatin, the mitochondria aggregated and tended to be surround the nucleus (Fig. S3).

In line with these findings, we examined the early embryo development potential of these oocytes. The matured oocytes were fertilized in vitro by intracytoplasmic sperm injection followed by assessment of early embryo development. As expected, high-dose cisplatin inhibited the development of early embryo development, as it inhibited oocyte maturation. Of note, although low-dose cisplatin did not affect the rates of oocyte maturation or pronuclei formation, the treatment significantly arrested embryos at the 2-cell stage (Fig. 4i). These findings suggested that despite the absence of obvious effects on oocyte morphology and maturation, a low dose of cisplatin caused a substantial change in intracellular ROS and mitochondrial membrane potential, and weakened early embryo development competence.

### Cisplatin exposure alters the profile of core oocyte mitochondrial proteins

**Fig. 5:**
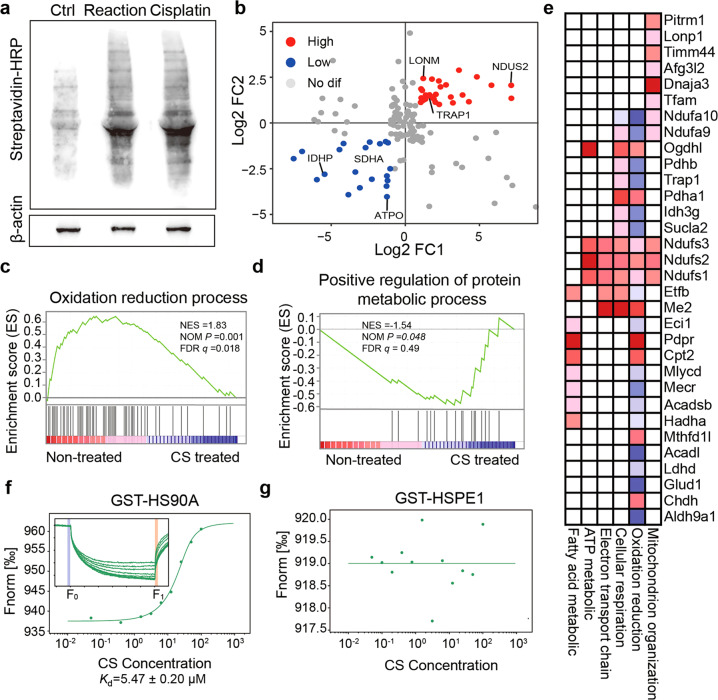
Characterization of dynamics in cisplatin-treated oocyte mitochondrial proteome. **a** Western blotanalysis of biotinylated proteins from oocytes with or without cisplatin treatment. Following the Mito-APEX2reaction, cisplatin-treated and non-treated oocyte lysates were run on SDS-PAGE and analyzed by streptavidin blotting. Oocytes expressing THIOM-EGFP served as the negative control. **b** The log2 of foldchange (Log2 FC) of oocyte mitochondrial protein quantitative values in comparison of cisplatin-treated versus untreated reacted oocytes. Proteins with higher abundance are colored red and lower abundance are colored blue, others are gray. **c**, **d** Gene Set Enrichment Analysis (GSEA) of mitochondrial proteins in cisplatin-treated and non-treated oocytes. GSEA enrichment plots of oxidative reduction (**c**) and positive regulation of protein metabolic process (**d**) showed that the abundance of mitochondrial proteins was altered after cisplatin treatment. NES, normalized enrichment score; NOM, nominal; FDR, false discovery rate. **e** Leading-edge analysis by GSEA to identify core proteins in enriched biological processes in cisplatin treated or non-treated oocyte mitochondrial proteomes. The color key from blue to red represents the protein expression level from low to high. **f**, **g** The in vitro-binding affinity between cisplatin and GST-HS90A or HSPE1 was tested by MST assay. Inset, thermophoretic movement of fluorescently labeled proteins. Fnorm = *F*_1_/*F*_0_ (Fnorm: normalized fluorescence; *F*_1_: fluorescence after thermodiffusion; *F*_0_: initial fluorescence or fluorescence after T-jump). *K*_d_, dissociation constant.

The APEX2 proximity labeling system also enabled the capture of oocyte mitochondrial proteomic remodeling under pharmacological treatment conditions in real time. Thus, to gain further insight into the effects of cisplatin on mitochondria, we employed the APEX2 proximity biotinylation system in cisplatin-treated oocytes. Considering that a low dose of cisplatin was sufficient to induce mitochondrial dysfunction, we cultured oocytes expressing THIOM-APEX2-EGFP with 1 μM cisplatin and performed the Mito-APEX2 reaction. The banding pattern of proteins biotinylated by APEX2 in cisplatin-treated oocytes was similar to that of non-treated reaction oocytes (Fig. 5a). However, quantitative proteomic analyses revealed alterations in the abundance of mitochondrial proteins after cisplatin treatment. Comparison of the 158 high-confidence oocyte mitochondrial proteins between cisplatin-treated and non-treated oocytes revealed that 58 were differentially expressed (Fig. 5b, Table S5). Specifically, 27 proteins were downregulated under cisplatin treatment, including SDHA, ATPO, and IDHP, which provide essential contributions to ATP synthesis (Fig. 5b), and 31 of these proteins were upregulated, including LONM, NDUS2, and TRAP1. As a component of the pro-survival mitochondrial pathway, TRAP1 is a mitochondrial chaperone protein that protects cells from DNA damage and apoptosis induced by oxidants or several other stress conditions [32]. We thus hypothesized that the higher abundance of TRAP1 might be responsible for immature oocyte survival after cisplatin treatment.

To analyze how protein sets changed in response to cisplatin treatment, we performed gene set enrichment analysis (GSEA). Through the integrated analysis of the statistical enrichment score and *P* value, we found that the qualitative abundance of proteins associated with the processes of “oxidation reduction”, “mitochondrion organization”, and “cellular respiration” decreased in cisplatin-treated oocytes (Fig. 5c, S4a–d). In comparison to non-treated reaction oocytes, proteins were significantly enriched in “positive regulation of protein metabolism” and “macromolecule catabolic” processes, which coincided with the stress response to cisplatin treatment (Fig. 5d, S4e–f). Leading-edge analysis further suggested that a variety of proteins contributed to the core enrichment of these main biological processes (Fig. 5e). These analyses indicated that cisplatin treatment altered the accumulation of mitochondrial proteins, resulting in the impairment of mitochondrial function. These altered proteins could be potential candidates for assessing mitochondrial quality upon different treatments.

We also paid attention to the effects of cisplatin on specific mitochondrial proteins in oocytes. For HS90A, we assumed that cisplatin would interact with HS90A and affect mitochondrial function. We thus expressed and purified the GST-HS90A protein in vitro (Fig. S4g), and tested the binding affinity with cisplatin by microscale thermophoresis (MST) assay. The known mitochondrial protein HSPE1 (GST-HSPE1), actin protein, and GST peptide were used as negative controls. Cisplatin had low binding affinity for HSPE1 (Fig. 5g), as well as actin or GST peptide (Fig. S4h, i). Notably, cisplatin bound to GST-HS90A with a dissociation constant (*K*_d_) value of 5.47 ± 0.20 μM (Fig. 5f). These results suggested that cisplatin preferentially binds a set of mitochondrial proteins and affects mitochondrial function.

## Discussion

Explorations of the molecular processes of oocytes have benefitted from single-cell multi-omics sequencing studies that have reported the transcriptome, DNA methylome, and chromatin accessibility of oocytes [25, 33]. Other recent studies have characterized the human oocyte proteome and identified a large number of proteins that are differentially expressed among oocytes at different developmental stages [34, 35], although attempts at proteomic profiling of oocyte organelles have been unsuccessful thus far. Mitochondria are energy-producing organelles that are required for numerous cellular processes in all types of eukaryotes [28]. Mitochondria are believed to play especially important roles in oocytes due to their substantial number in these large-volumed female gametes [36]. It is estimated that the thousands of mitochondria in oocytes are formed from a few hundred present in primordial germ cells [37, 38]. Of note, these mitochondria are transcriptionally silent, in strong contrast to the mitochondria in somatic cells [39, 40], emphasizing the unique contribution of proteins in oocyte mitochondria. Thus, proteomic profiling of oocyte mitochondria will allow us to identify the core factors and signaling pathways orchestrating key events in the germ cells, and will offer a valuable resource for further mechanistic studies and applications. In this study, we implemented a proximity labeling strategy to generate high-confidence mitochondrial proteomic maps of mouse GV oocytes. Using an engineered APEX2 fused with the mitochondrial matrix protein THIOM that targeted the mitochondria of live oocytes, we labeled endogenous mitochondrial proteins with BP for subsequent purification and identification. In our study, we identified 158 high-confidence proteins specific to oocyte mitochondria, thus overcoming limitations imposed by non-physiological purification of lysed samples. Moreover, this strategy effectively identified mitochondrial proteins using a relatively small number of oocytes.

When compared to previous studies [27], we found that Mito-targeted APEX2 protein labeling in oocyte mitochondria facilitated the identification and localization patterns of a substantially greater proportion of captured proteins. Functional analysis confirmed that these cellular components clustered primarily into “mitochondrial matrix”, “mitochondrial inner membrane”, “oxidoreductase complex”, “mitochondrial nucleoid”, and “mitochondrial respiratory chain”. These validated mitochondrial proteins function as intrinsic components of mitochondrial structure or as enzymes involved in metabolism and energy production. A previous study reported an interactome of THIOM in mouse organs (lung, kidney, skeletal muscle, and brain) consisting of 52 interacting mitochondrial proteins [41]. Among these 52 proteins (including mitochondrial proteins from lung, kidney, skeletal muscle, and brain tissues), 20 were also found in our oocyte sample. Analysis of the 20 THIOM-interacting proteins in oocytes suggests that they mainly play roles in citrate metabolic processes, oxidative stress and electron transport chain. For example, mitochondrial aconitase (ACO2) is a reversible enzyme that catalyzes the conversion of citrate to isocitrate in the citric acid cycle [42]. NDUFS1, 2, and 3 are major subunits of mitochondrial respiratory chain complex I [43], and the interactions of THIOM with NDUFS1, 2 and 3 imply a possible involvement of THIOM in maintaining the functional and structural integrity of complex I. Our study also identified various new proteins that localized specifically in oocyte but not in somatic cell mitochondria, such as heat shock protein 90α (HS90A), eukaryotic elongation factor 2 (EF2), and receptor for activated C kinase 1 (RACK1). The identification of these proteins may provide valuable insights for improving our understanding of oocyte biology and oocyte quality. For instance, HS90A belongs to the HSP90 family, members of which are evolutionarily conserved molecular chaperones required for the stability and function of more than hundreds of proteins [44, 45]. In somatic cells, HS90A is localized in the cytoplasm and nucleus, and its deregulation has been proven to be correlated with various diseases [46]. The retention of HS90A in oocyte mitochondria implies an additional role in germ cells. Due to the long period arrest of immature oocytes, the proteins in oocyte mitochondria have prolonged exposure to the electron transport chain, which continuously generates reactive oxygen species (ROS). Thus, the localization of molecular chaperones to the mitochondria would be necessary for oocyte protection.

Another goal of this study was to evaluate the impacts of cisplatin on mitochondrial proteomic dynamics in oocytes. It is well known that cisplatin causes adverse effects on oocyte quality in female cancer patients during chemotherapy. However, how and to what extent cisplatin damages the germ cells is unclear. Most previous studies were limited to the analysis of genomic damage in the nucleus [47–49] since the cytotoxic effect of cisplatin was thought to be mediated primarily by the formation of inter-strand and intra-strand DNA adducts, which disrupt cellular transcription and replication [50]. Nevertheless, ‘omics’-based shifts in the cytoplasm, which stores essential maternal materials for oocyte maturation and early embryonic development, and especially for the mitochondria, are absent. In this study, we proved that a low dose of cisplatin referenced from clinical chemotherapy is sufficient to lead to a significant shift in the mitochondrial proteome compared to the control group, even though this concentration did not lead to morphological changes and or maturation decreases of GV oocytes. These oocytes contained impaired mitochondria marked by elevated ROS and abnormal mitochondrial membrane potential. Protein profiling demonstrated that the abundances of 58 mitochondrial proteins changed following cisplatin treatment, especially proteins involved in stress responses such as positive regulation of protein metabolism (e.g., TRAP1, NDUS2, and TBCD1). Oxidative stress can lead to cytotoxic accumulation of misfolded or oxidized aberrant proteins, which in healthy cells are degraded by proteases in the mitochondrial matrix [51]. We observed that cisplatin treatment causes an increase in ROS levels within the oocyte, and the increased accumulation of mitochondrial proteases may serve as a compensation mechanism when deleterious levels of ROS are reached. Thus, dynamic changes in the mitochondrial proteome can facilitate further exploration of proteins participating in pro-survival processes in immature oocytes and establish a comprehensive evaluation system for evaluating chemotherapeutic side effects on oocyte quality.

## Supplementary information

Supplementary information figS1-S4

Table S1

Table S2

Table S3

Table S4

Table S5
