# Supplementary material for: Mitochondrial proteome of mouse oocytes and cisplatin-induced shifts in protein profile
Source: Acta Pharmacol Sin. 2021 May 20;42(12):2144–54. doi: 10.1038/s41401-021-00687-4 (PMC8632880; doi:10.1038/s41401-021-00687-4)
Supplement: Supplementary file 1 — Supplementary information figS1-S4 [file 41401_2021_687_MOESM1_ESM.pdf]

Figure S1

Gene Symbol	Mass(kDa)*	Location	Oocyte 1 (RPKM)	Oocyte 2 (RPKM)
Txn2	18.255	Matrix	93.8	64.7
Ccdc90b	29.597	Inner membrane	89	78.1
Slc25a31	35.258	Inner membrane	110.8	71
Idh3a	39.639	Matrix	62.5	54.4
Acat1	44.816	Matrix	100.6	131.7
Uqcrc2	48.235	Inner membrane	132.2	87
Trnt1	49.895	Matrix	64.3	58.6
Cs	51.737	Matrix	108.5	96.4
Atp5f1b	56.300	Inner membrane	229.7	234.4
Sdha	72.585	Inner membrane	97.5	96.8

* Ranked by the mass of proteins

Fig. S1 APEX2 fusion protein screening for mitochondrial protein localization in mouse oocytes. APEX2 with an EGFP tag was fused to 10 different candidate mitochondrial proteins predicted to guide APEX2 to mitochondria.

Figure S2

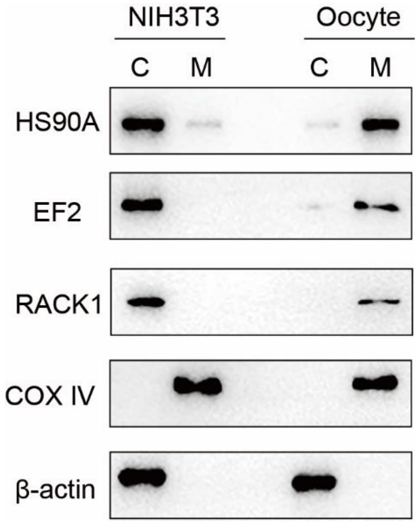

Fig. S2 Cytosolic and mitochondrial fractions of NIH3T3 cells and oocytes were separated and analyzed by western blotting with anti-HS90A, EF2 or RACK1 antibody. Mitochondrial protein Cox IV and cytosolic protein β-actin were used as loading controls. C: cytosolic fraction; M: mitochondrial fraction.

Figure S3

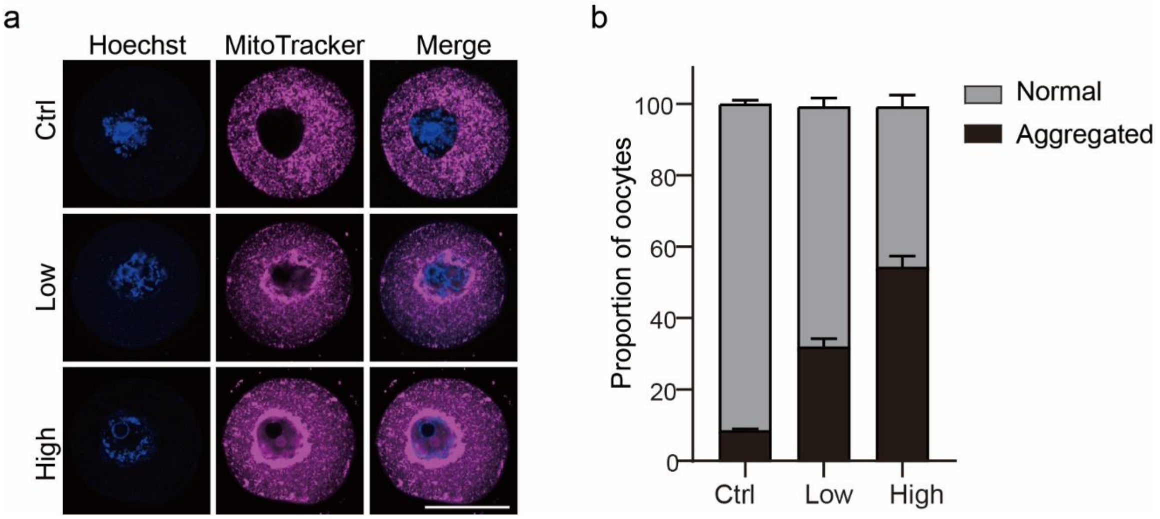

Fig. S3 The effect of cisplatin on mitochondrial distribution. **a** Representative images of mitochondria in control and cisplatin-treated groups. GV oocytes were treated with low and high dosages of cisplatin for 4 h and stained with MitoTracker to observe mitochondrial distribution in oocytes. MitoTracker (magenta), DNA (blue). Scale bar, 50 µm. **b** Histogram shows the proportion of abnormal mitochondrial distribution in control and cisplatin-treated groups. The data represent the mean ± SD.

Figure S4

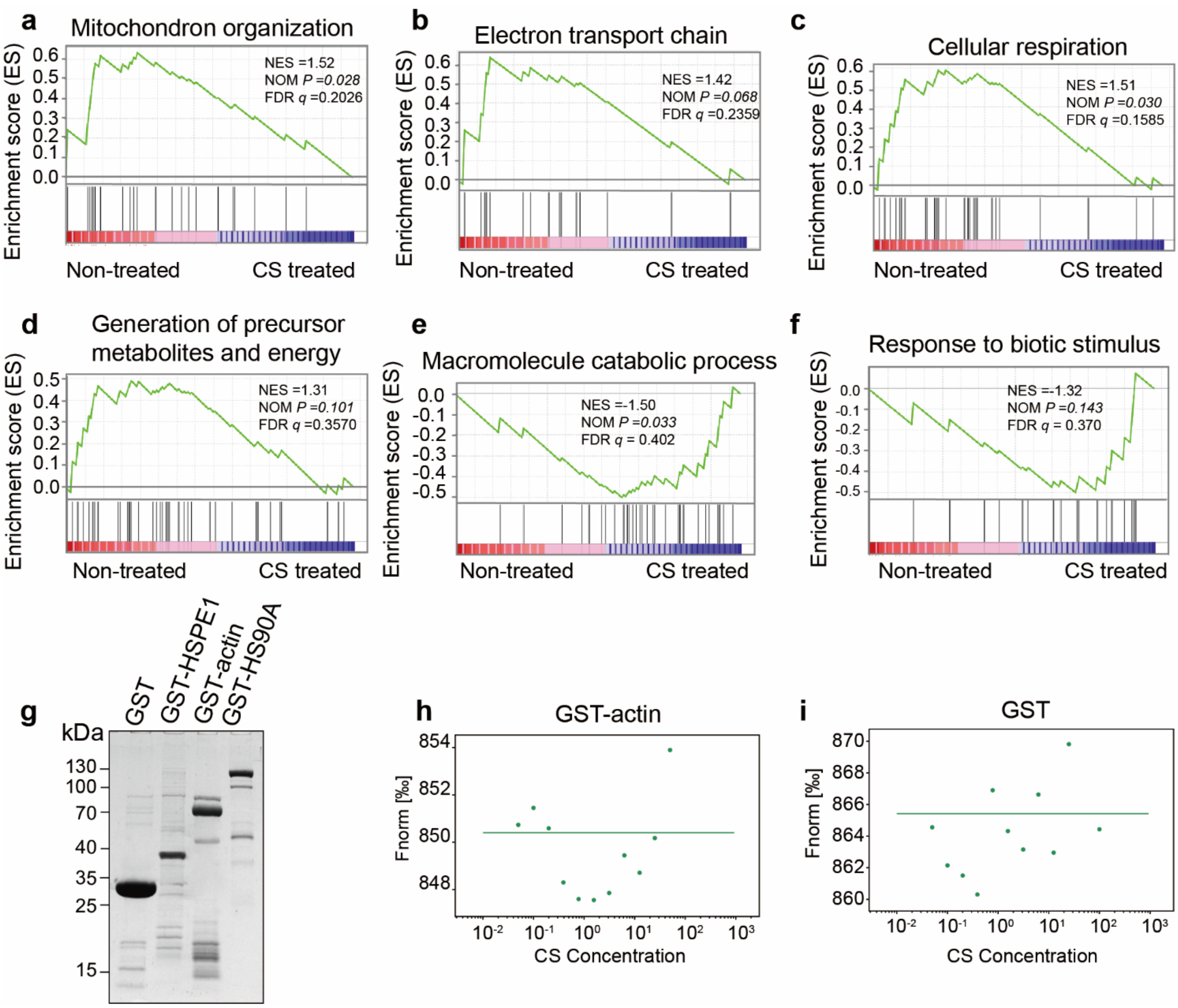

Fig. S4. a-f Gene Set Enrichment Analysis (GSEA) of mitochondrial proteins in cisplatin-treated and non-treated oocytes, related to Fig. 5**c, d**. GSEA enrichment plots of mitochondrion organization (**a**), electron transport chain (**b**), cellular respiration (**c**), generation of precursor metabolites and energy (**d**), macromolecule catabolic (**e**) and response to biotic stimulus (**f**) are shown. NES, normalized enrichment score; NOM, nominal; FDR, false discovery rate. **g** Coomassie brilliant blue staining of proteins used for MST microscale thermophoresis (MST) assay. **h, i** The in vitro-binding affinity between cisplatin and GST-actin or GST was tested by MST assay.
